# Acquisition of different transcriptional shear mRNA and biological function of porcine interleukin 18 binding protein in PRRSV infection

**DOI:** 10.1128/mbio.00640-24

**Published:** 2024-05-10

**Authors:** Hong-zhe Zhao, Chun-yu Liu, Qian-jin Song, Hao Guo, Yong-jun Wen, Feng-xue Wang

**Affiliations:** 1 Key Laboratory for Clinical Diagnosis and Treatment of Animal Diseases of Ministry of Agriculture, College of Veterinary Medicine, Inner Mongolia Agricultural University, Hohhot, China; 2 Medical Experiment Center, Inner Mongolia Medical University, Hohhot, China; 3 Yinchuan Animal Husbandry Technology Extension Service Center, Yinchuan, China; Catholic University of America, Washington, DC, USA; Boston Children's Hospital, Boston, Massachusetts, USA

**Keywords:** porcine interleukin-18 binding protein, porcine interleukin-18, porcine interferon-γ, porcine reproductive and respiratory syndrome virus, porcine alveolar macrophage

## Abstract

Interleukin-18 binding protein (IL-18BP), a natural regulator molecule of the pro-inflammatory cytokine interleukin-18 (IL-18), plays an important role in regulating the expression of the cellular immunity factor interferon-γ (IFN-γ). In a previous RNA-seq analysis of porcine alveolar macrophages (PAM) infected with the TIM and TJ strains of porcine reproductive and respiratory syndrome virus (PRRSV), we unexpectedly found that the mRNA expression of porcine interleukin 18-binding protein (pIL-18BP) in PAM cells infected with the TJM strain was significantly higher than that infected with the TJ strain. Studies have shown that human interleukin-18 binding protein (hIL-18bp) plays an important role in regulating cellular immunity in the course of the disease. However, there is a research gap on pIL-18BP. At the same time, PRRSV infection in pigs triggers weak cellular immune response problems. To explore the expression and the role of pIL-18BP in the cellular immune response induced by PRRSV, we strived to acquire the pIL-18BP gene from PAM or peripheral blood mononuclear cell (PBMC) with RT-PCR and sequencing. Furthermore, pIL-18BP and pIL-18 were both expressed prokaryotically and eukaryotically. The colocalization and interaction based on recombinant pIL-18BP and pIL-18 on cells were confirmed *in vitro*. Finally, the expression of pIL-18BP, pIL-18, and pIFN-γ was explored in pigs with different PRRSV infection states to interpret the biological function of pIL-18BP *in vivo*. The results showed there were five shear mutants of pIL-18BP. The mutant with the longest coding region was selected for subsequent functional validation. First, it was demonstrated that TJM-induced pIL-18BP mRNA expression was higher than that of TJ. A direct interaction between pIL-18BP and pIL-18 was confirmed through fluorescence colocalization, bimolecular fluorescent complimentary (BIFC), and co-immunoprecipitation (CO-IP). pIL-18BP also can regulate pIFN-γ mRNA expression. Finally, the expression of pIL-18BP, pIL-18, and pIFN-γ was explored in different PRRSV infection states. Surprisingly, both mRNA and protein expression of pIL-18 were suppressed. These findings fill the gap in understanding the roles played by pIL-18BP in PRRSV infection and provide a foundation for further research.

**IMPORTANCE:**

PRRSV-infected pigs elicit a weak cellular immune response and the mechanisms of cellular immune regulation induced by PRRSV have not yet been fully elucidated. In this study, we investigated the role of pIL-18BP in PRRSV-induced immune response referring to the regulation of human IL-18BP to human interferon-gamma (hIFN-γ). This is expected to be used as a method to enhance the cellular immune response induced by the PRRSV vaccine. Here, we mined five transcripts of the pIL-18BP gene and demonstrated that it interacts with pIL-18 and regulates pIFN-γ mRNA expression. Surprisingly, we also found that both mRNA and protein expression of pIL-18 were suppressed under different PRRSV strains of infection status. These results have led to a renewed understanding of the roles of pIL-18BP and pIL-18 in cellular immunity induced by PRRSV infection, which has important implications for the prevention and control of PRRS.

## INTRODUCTION

**Fig 1:**
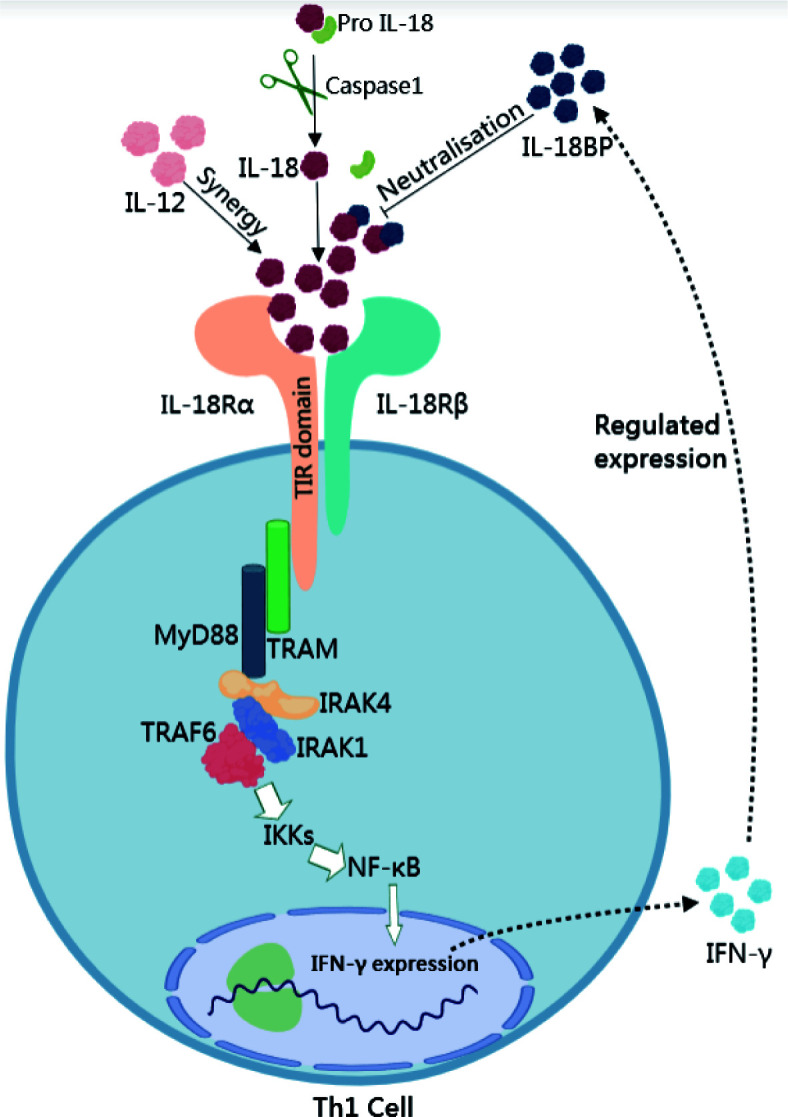
Biological functions of IL-18BP. IL-18BP binds to IL-18, thereby inhibiting the biological function of IL-18. IL-18 binding to the IL-18 receptor activates the MyD88-NF-kB pathway in concert with IL-12, which induces IFN-γ expression. Meanwhile, overexpression of IFN-γ induces IL-18BP expression. IL-18αR, interleukin-18 alpha receptor; IL-18βR, interleukin-18 beta receptor; MyD88, myeloid differentiation factor 88; TRAF6, TNFR-associated factor 6;TRAM, translocating chain-associated membrane protein; IRAK, interleukin receptor-associated kinase; NF-κB, nuclear factor-κ-gene binding.

Porcine reproductive and respiratory syndrome (PRRS) is an infectious disease caused by PRRSV, which was first reported in North America in 1987, and subsequently detected in Europe and Asia in 1990 (1–3). The virus is now endemic in most pig farming countries and is one of the main causes of economic losses in the pig industry. The economic cost of the disease to the U.S. farming industry is reported to be approximately $560 million per year, with weaned piglets and breeding stock accounting for 55% of the total cost (4). This compares to an average economic loss of €126 per sow during the PRRS outbreak in Europe (5). Typical immune features of PRRSV infection are a weak innate immune response, delayed appearance of neutralizing antibodies, and a weak cellular immune response (6–9). IFN-γ, a key driver of cellular immunity, orchestrates multiple protective functions to enhance the immune response to viral infection (10). However, infection with PRRSV or vaccination against PRRSV only induces a weak and non-specific IFN-γ response (11, 12). At the same time, the vaccine strain used in the United States does not provide sufficient heterologous protection, probably because it does not stimulate a sufficient interferon-gamma response (13). Studies have shown that IL-18 was identified as an IFN-γ inducer and that it acts synergistically with interleukin-12 (IL-12) to induce IFN-γ production by T cells and natural killer (NK) cells, suggesting that it plays an important role in the Th1 immune response (14). However, studies have shown that the activity of IL-18 is mainly regulated by its natural inhibitor IL-18BP, which has a high affinity for IL-18 and can effectively block the interaction between IL-18 and its receptor IL-18R, thereby inhibiting IL-18-induced secretion of IFN-γ from Th1 cells and decreasing IL-18 stimulated nuclear factor-κB (NF-κB) activity (Fig. 1) (15, 16). Meanwhile, induction of endogenous IL-18BP expression and exogenous injection of recombinant IL-18BP protein were both effective in inhibiting IL-18 activity (17–19). A study reported in the journal *Nature* showed that IL-18 was expected to be used in the clinical oncology treatment of human medicine for tumors. However, the effect was not significant. It was later discovered that IL-18BP, acting as a secretory immune checkpoint, restricts the anti-tumor activity of IL-18. The invention of IL-18 with “decoy resistance” (DR-18) overcome this restriction and demonstrated powerful anti-tumor activity (20). Meanwhile, research in AIDS (HIV) infection has suggested that an imbalance between IL-18 and its antagonist IL-18BP occurs in the circulation of HIV-infected individuals, whereas this imbalance is absent in long-term non-progressors of HIV infection (LTNPs) and maintains normal levels of IL-18BP in the circulation (21). This suggests that IL-18BP determines disease progression. The role played by pIL-18BP in PRRSV infection is currently unknown. By checking the GenBank database, we found that there is only the computer-predicted pIL-18BP gene, which has not been experimentally verified. Therefore, the present study was performed to obtain the experimentally validated pIL-18BP gene from PAM cells and to investigate its biological function in cellular immunity induced by PRRSV infection to provide new ideas for the prevention and treatment of PRRSV.

## MATERIALS AND METHODS

### Viruses and cells

The HP-PRRSV TJ strain (NCBI Genbank; accession number: EU860248) was proliferated in Marc-145 cells. The PRRSV TJM vaccine strain is an attenuated strain obtained by passaging the TJ strain on Marc-145 cells (deletion of 120aa at positions 628–747 of NSP2). PRRSV NADC34-like medium virulence strain was isolated from Tongliao City, Inner Mongolia (NCBI Genbank; accession number: OR753369). PAM and PBMC were prepared according to previous studies (22, 23).

### Sample preparation

The resuscitated PAM was inoculated into a six-well cell plate and changed to a maintenance solution after 12 h. Subsequently, the cells were inoculated with the PRRSV TJM strain at a ratio of MOI = 0.1. After 48 h, the cells were collected and RNA was extracted using an RNA extraction kit. Finally, the cells were reverse-transcribed into cDNA using a reverse-transcription kit and set aside.

### Acquisition of porcine IL-18 and IL-18BP genes

Primers were designed using Primer Premier 6.0 based on the predicted pIL-18BP gene sequence, which was published in GenBank. The details of all primers involved in this study are shown in Table 1. The pIL-18 and pIL-18BP-X1, X2 genes were amplified using PCR with the pIL-18 and pIL-18BP-X1, X2 primers. The reaction system consisted of 50 µL, with Kod One 25 µL, upstream and downstream primers 1.5 µL each, ddH_2_O 21 µL, and template 1 µL. The PCR was programmed as follows: pre-denaturation at 94°C for 2 min, denaturation at 98°C for 10 s, annealing at 59°C for 5 s, extension at 68°C for 10 s, total extension at 68°C for 10 min and 35 cycles. Finally, the PCR product was ligated into the pMD-19T vector and sequenced. The correct sequences were submitted to the GenBank database.

**TABLE 1:** Primer information^*a*^

Primer name	Sequences	Restriction endonucleases	GenBank number of the primer design
pIL-18	F: **CG**GAATTCATGGCTGCTGAACCGGAAGA	*Eco*RI	AF191088.1
	R: **CC**CTCGAGGTTCTTGTTTTGAACAGTG	*Xho*I	
	F: **CG**GAATTCGGATGGCTGCTGAACCGGAAGA	*Eco*RI	
	R: **GG**GGTACCGTTCTTGTTTTGAACAGTG	*Kpn*I	
pIL-18BP-X1	F: CGGAATTCATGACCAGGAGGCAGAACTGG	*Eco*RI	XM_003129618.5
	R: **CC**CTCGAGTGCGCGCCCGGCGTGGGCC	*Xho*I	
pIL-18BP-X2	F: **CG**GAATTCATGACCAGGAGGCAGAACTGG	*Eco*RI	XM_003129617.5
	R: **CC**CTCGAGGGGCCGAGGCAGGAGGGCCG	*Xho*I	
	F: **CCC**AAGCTTATGACCAGGAGGCAGAACTGG	*Hin*dIII	
	R: **GC**TCTAGAGGGCCGAGGCAGGAGGGCCG	*Xba*I	
Q-pIL-18	F: ACGATGAAGACCTGGAATC	*—*	AF191088.1
	R: AACAGTCAGAATCAGGCATA	*—*	
Q-pIL-18BP-X2	F: CGCCACGCCAACTTCTCCT	—	XM_003129617.5
	R: CCTGTTCCTTCTCGTTTCCTG	—	
Q-β-Actin	F: CTGGCATTGTCATGGACTCT	—	XM_003357928.4
	R: GCGATGATCTTGATCTTCAT	—	
Q-pIFN-γ	F: CTTTGCGTGACTTTGTGTT	—	NM_213948.1
	R: CCATTAGGTACATCTGAGGTA	—	

^*a*^ Boldfacing indicates protective bases; underlining indicates enzymatic sites.

### Validation of previous RNA-seq results

Since the previous RNA-seq results of our group indicated that the mRNA expression of the pIL-18BP gene induced by the TJM strain was higher than that of the TJ strain, the results were first verified *in vitro* (24). The resuscitated PAM was inoculated into six-well plates (1 × 10^6^/well) and incubated for 12 h at 37°C with a 5% CO_2_ concentration in an incubator. Then the cells were inoculated with the TJ and TJM strains at an MOI of 0.1. The cells were collected at 3, 6, 12, and 24 h post-infection, and stored in the refrigerator at −80°C for future use. Total RNA was extracted and reverse-transcribed into cDNA. Finally, the expression of the pIL-18BP and pIFN-γ mRNA was verified using qPCR methods with Q-pIL-18BP-X2, Q-pIFN-γ, and Q-β-Actin primers.

### Vector construction

Recombinant plasmids pCAGGS-pIL-18 and BIFC-VC155-pIL-18 were constructed by ligating the pIL-18 gene to the eukaryotic expression vectors pCAGGS and pBIFC-VC155 using enzymatic ligation technology. Then the pIL-18BP gene was ligated into the eukaryotic expression vectors pcDNA3.1-3×flag and pBIFC-VN173 to construct the recombinant plasmids pcDNA3.1-3×flag-pIL-18BP and pBIFC-VN173-pIL-18BP. Next, the pIL-18 and pIL-18BP genes were ligated into the prokaryotic expression vector pET28a to construct recombinant plasmids pET28a-pIL-18 and pET28a-pIL-18BP, respectively. Finally, all the correctly sequenced recombinant plasmids were stored for spare use.

### Plasmid validation

First, pCAGGS-pIL-18, pcDNA3.1-3×flag-pIL-18BP, and pBIFC-VC155-pIL-18 recombinant plasmids were transfected into HEK-293FT cells that had already grown a full monolayer, respectively. After 48 h post-transfection, cells were washed once with PBS. Then, cell lysate containing 1% PMSF was added, and proteins were lysed on ice for 10 min. The lysate was then collected and centrifuged at 12,000 × *g* at 4°C for 20 min, and the supernatant was collected and added with a final concentration of 1 × protein buffer and denatured in a metal bath at 100°C for 10 min. Next, a western blot (WB) was performed to verify the proper functioning of the eukaryotic plasmids. Finally, we induced expression and purified the pET28a-pIL-18 and pET28a-pIL-18BP in *E. coli* BL21. A western blot was applied to verify the expression of the protein. The protein pIL-18BP purified by prokaryotic expression was sent to Beijing Huada Biological Co. for mass spectrometry identification.

### Functional validation of pIL-18 and pIL-18BP

#### Immunofluorescence

First, we co-transfected Vero E6 cells with pCAGGS-pIL-18 and pcDNA3.1-3×flag-pIL-18BP plasmids. After 48 h, the cells were fixed with 4% paraformaldehyde and incubated at 37°C for 1 h with 5% BSA. Then, they were incubated with antibodies to pIL-18 (R&D, USA) and flag (Sigma, USA). Subsequently, AF488 and AF647 (Abcam, England) labeled secondary antibodies were incubated after washing with PBS. The cells were washed again with PBS and incubated with DAPI at room temperature. Finally, the results were observed under a laser confocal microscope after a final PBS wash.

#### BIFC

HEK-293FT cells that had grown all over the monolayer were replaced with fresh complete medium. HET-293FT cells were cotransfected with pBIFC-VN173-pIL-18BP and pBIFC-VC155-pIL-18 eukaryotic expression plasmids using liposome assay. The results were observed under a laser confocal microscope 48 h after transfection. The plasmids pBIFC-VN173-pIL-18BP and pBIFC-VC155 were also co-transfected in 293 FT cells as a negative control.

#### Co-IP

Vero E6 cells were co-transfected with the pcDNA3.1-3×flag-pIL-18BP plasmid and the pCAGGS-pIL-18 plasmid as the test group. Vero E6 cells were also co-transfected with the pcDNA3.1-3×flag and pCAGGS-pIL-18 plasmid as the control group. Next, 100 µL of anti-flag M2 agarose affinity gel solution was taken and washed with PBS. 293T cells, which had been transfected for 36 h, were then lysed on ice using IP mild lysate containing 1% PMSF. The lysed cell supernatant was added to the anti-flag M2 agarose affinity gel solution and placed in a rotary mixer. It was incubated overnight at 4°C. Finally, the washed precipitate was subjected to western blot analysis. Primary antibodies were incubated with the IL-18 antibody (R&D, USA) and flag antibody (Sigma, USA), respectively.

#### Effect of different IL-18BP protein concentrations on pIFN-γ expression

Porcine peripheral blood lymphocytes were diluted to a concentration of 2 × 10^6^ cells/mL using RPMI 1640, which contained 5 µg/mL ConA and 10% FBS. These cells were then added to 96-well plates at a volume of 100 µL per well, resulting in a total of 2 × 10^5^ cells/well, and the plates were incubated in an incubator for 2 h. According to the previous studies on IL-18 functionality, it is known that the synergistic action of IL-18 and IL-12 is necessary to effectively induce IFN-γ production *in vitro* (14). Therefore, in this study, recombinant pIL-18 and pIL-12 (R&D, America) at concentrations of 500 ng/mL and 100 ng/mL, respectively, were co-incubated with different concentrations of pIL-18BP (1000, 750, 500, and 250 ng/mL) for 2 h at room temperature. After pIL-18, pIL-18 +pIL-12, pIL-12, and blank control were set up at the same time, the incubation was carried out at 37℃ for 3 h, 6 h, 12 h, and 24 h. Finally, cells from each time period were collected separately and subjected to mRNA extraction. The extracted mRNA was then reverse-transcribed into cDNA, and the gene expression levels of pIFN-γ were determined using qPCR.

#### Expression of pIL-18, pIL-18BP, and pIFN-γ in peripheral blood of pigs in different PRRSV infection states

Twelve healthy piglets, aged between 4 and 6 weeks, were screened using qPCR and ELISA to ensure they did not have antigenic antibodies to PRRSV, classical swine fever virus (CSFV), pseudo rabies virus (PRV), and porcine circovirus 2 (PCV2). These piglets were then randomly divided into four groups, with each group consisting of three piglets. The test groups (TJ, TJM, and NADC34 groups) were challenged with a dose of 3 × 10^6^ TCID_50_ of the TJ, TJM, and NADC34 strains, respectively. The control group was immunized against DMEM. The Challenge was administered through nasal spray and intramuscular injection in the neck. Anticoagulant and procoagulant blood samples were collected at 0, 3, 5, 7, 10, and 14 days after challenge (dpc). Total RNA was extracted from the collected anticoagulated blood using Trizol Reagent. The relative expression of pIL-18, pIL-18BP, and pIFN-γ in porcine whole blood was identified with relative quantitative PCR. In addition, pIL-18 ELISA (Thermo, America) and pIFN-γ ELISA (Meimian, China) assay kits were used to detect the protein expression of pIL-18 and pIFN-γ.

### Statistical analysis

All data in this study were set up with both within-group and between-group replications and were analyzed for significance using one-way ANOVA with SPSS 2.0. In addition, all data were expressed as mean ± SD.

## RESULTS

### Gene acquisition and submission to the GenBank database

After sequencing, we successfully cloned one pIL-18 gene and five pIL-18BP transcriptional mutants and named the five mutants X1–X5. The nucleotide sizes of the five pIL-18BP mutants were 620 bp, 573 bp, 710 bp, 240 bp, and 461 bp, respectively. Where the X1 and X3 sequences are compared, a 90 nucleotide deletion in the middle part of the sequence can be found. When X2, X4, and X5 were compared, we found that X4 and X5 had different degrees of deletion compared to X2, and the coding region of X2 belonged to the longest mutant among them, and it had a complete protein after translation without the phenomenon of termination codon, so X2 was used in this study as the gene for the subsequent functional study. We have submitted five mutants to GenBank (GenBank numbers: OL677567–OL677571).

### Relative expression of pIL-18BP and pIFN-γ on PAM and PBMC infected with PRRSV virus

**Fig 2:**
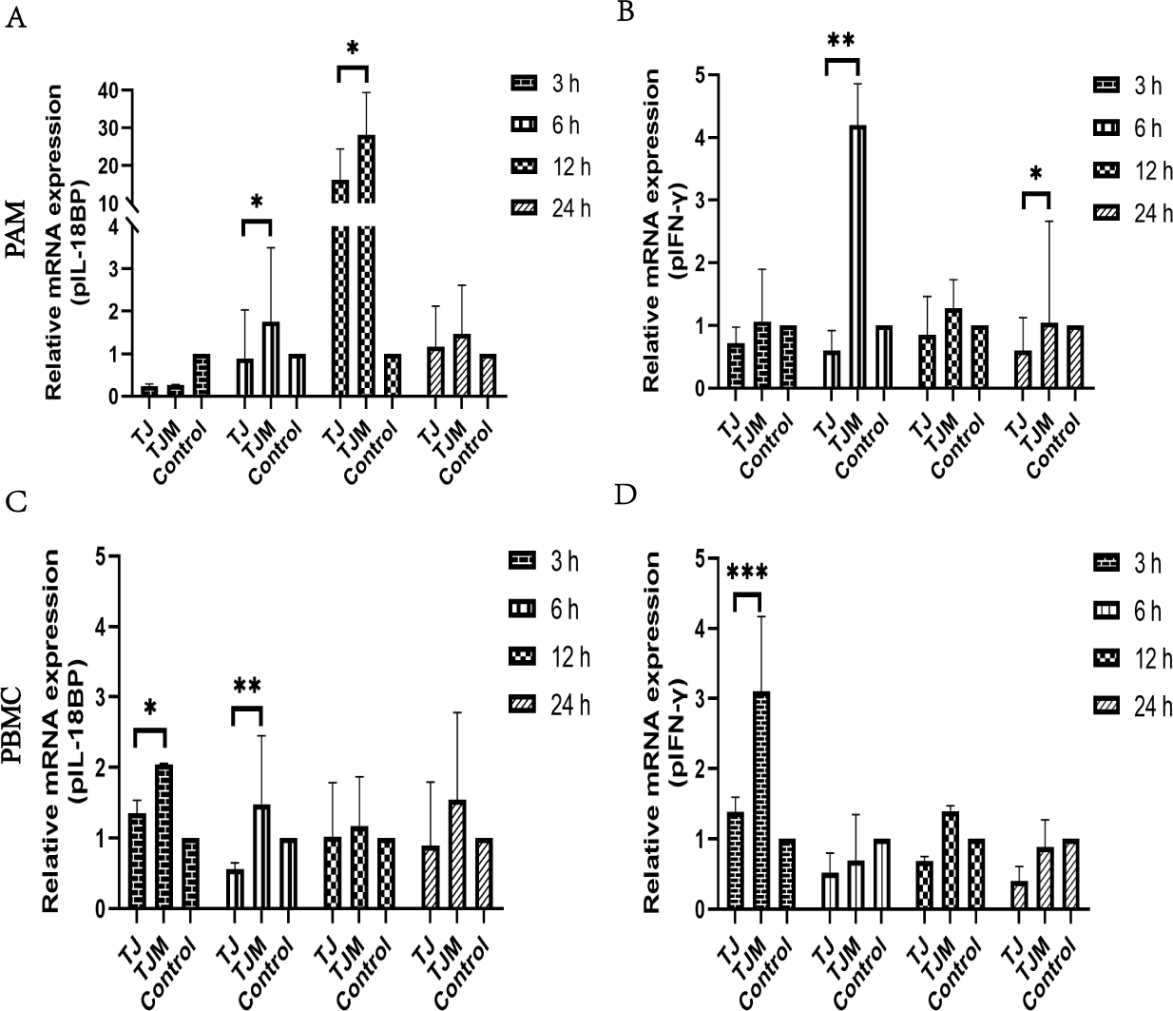
Relative expression results of pIL-18BP and pIFN-γ. (**A and B**) The mRNA on PAM cells. (**C and D**) The mRNA on PBMC cells. The data were expressed as mean ± SD. Asterisks indicate the statistical significance: *P, 0.05; **P, 0.01; ***P, 0.001.

When the PAM was infected with PRRSV TJ and TJM strains for 3 h, the mRNA expression of pIL-18BP was lower than that of the control group. This may be due to the early expression of pIL-18. However, at 6, 12, and 24 h, the expression of pIL-18BP was higher in PAM infected with TJM compared to the TJ-infected PAM. This difference was especially pronounced at 12 hpi (Fig. 2A). Furthermore, we found that the relative expression of pIL-18BP mRNA was higher in the PBMC infected with TJM strain at all four time points compared to the TJ-infected groups and control groups on PBMC (Fig. 2C). Meanwhile, the relative expression of pIFN-γ was higher in the TJM-infected groups than the TJ-infected groups and showed a dynamic distribution trend (Fig. 2B and D). Furthermore, we found that the expression of pIFN-γ was inhibited after the PAM infected with the TJ strain. It was lower than that of the control group at 6 h, 12 h, and 24 h in PBMC (Fig. 2B and D). This result supports the previous finding that the PAM infected with a weakly virulent TJM strain, as observed in the RNA-Seq analysis, exhibits significantly higher expression than the PAM infected with a strongly virulent TJ strain. This similar result is also observed on PBMC.

### Validation of eukaryotic and prokaryotic expression

**Fig 3:**
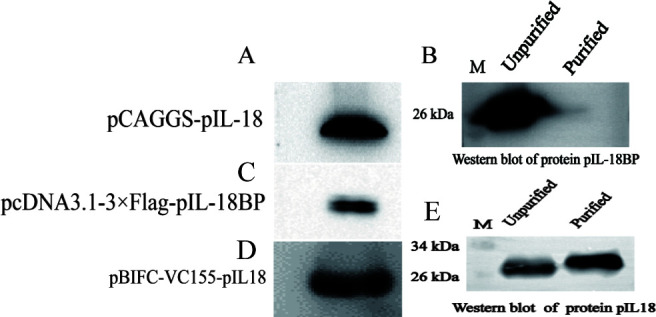
Western blot identification results of eukaryotic and prokaryotic expression. (A, C, and D) Identification of eukaryotic expression of pIL-18 and pIL-18BP. (B and E) Identification of prokaryotic expression of pIL-18 and pIL-18BP.

The pIL-18 and pIL-18BP genes were successfully ligated into eukaryotic and prokaryotic expression vectors, and they were sequenced correctly. The WB results clearly indicate that the two target proteins are more homogeneous, suggesting that pIL-18 and pIL-18BP proteins have been expressed and that the three recombinant plasmids are functioning properly (Fig. 3A, C and D). Meanwhile, we transformed the prokaryotic expression vectors pET28a-pIL-18 and pET28a-pIL-18BP into *E. coli* BL21 sensory state and performed prokaryotic expression. We then carried out affinity purification using a Ni column. The purified proteins were subsequently subjected to WB validation, which confirmed the successful expression and purification of pIL-18 protein was successful, while pIL-18BP protein indirectly proved that expression purification was successful (Fig. 3B and E). Finally, we identified the expressed pIL-18BP protein through mass spectrometry, and the results confirmed the successful expression of the pIL-18BP protein.

### pIL-18BP and pIL-18 can interact directly and co-locate in the cytoplasmic region of cells

**Fig 4:**
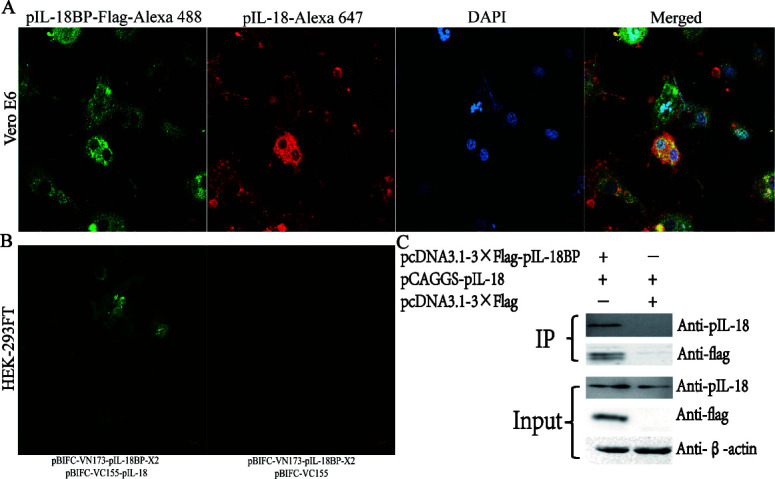
Identification of pIL-18 and pIL-18BP protein interactions. (**A**) Fluorescence co-localization. (**B**) Bimolecular fluorescence complementation. (**C**) Co-immunoprecipitation

We used three colors to label the different proteins: pIL-18BP was labeled green, pIL-18 was labeled red, and the nucleus was labeled blue. Co-transfection was performed on Vero E6 cells, and IFA identification was conducted at 48 h post-transfection. The results showed that both pIL-18 and pIL-18BP proteins were expressed in the cytoplasm and overlapped with each other, suggesting a potential interaction between the two proteins (Fig. 4A). Subsequently, BIFC validation was performed. The results revealed significant green fluorescence in the cytoplasm of cells co-transfected with BIFC-VN173-pIL-18BP and BIFC-VC155-pIL-18, while no green fluorescence was observed in control cells co-transfected with BIFC-VN173-pIL-18BP-X2 and BIFC-VC155. This suggests that there is an interaction between the pIL-18BP protein and the pIL-18 protein (Fig. 4B). To further confirm the direct interaction, we co-transfected pcDNA3.1×flag-pIL-18BP and pCAGGS-pIL-18 in Vero E6 cells as the experimental group. We also co-transfected pCAGGS-pIL-18 and pcDNA3.1×flag as the control group for CO-IP validation. The results demonstrated that the beads using anti-flag tags were able to capture pIL-18BP and also pull down pIL-18. By contrast, no pIL-18BP was detected in the control group, and pIL-18 was not pulled down. This provides further evidence of an interaction between pIL-18BP and pIL-18 (Fig. 4C).

### The pIL-18BP protein affects pIFN-γ mRNA expression with dose-dependent *in vitro*

**Fig 5:**
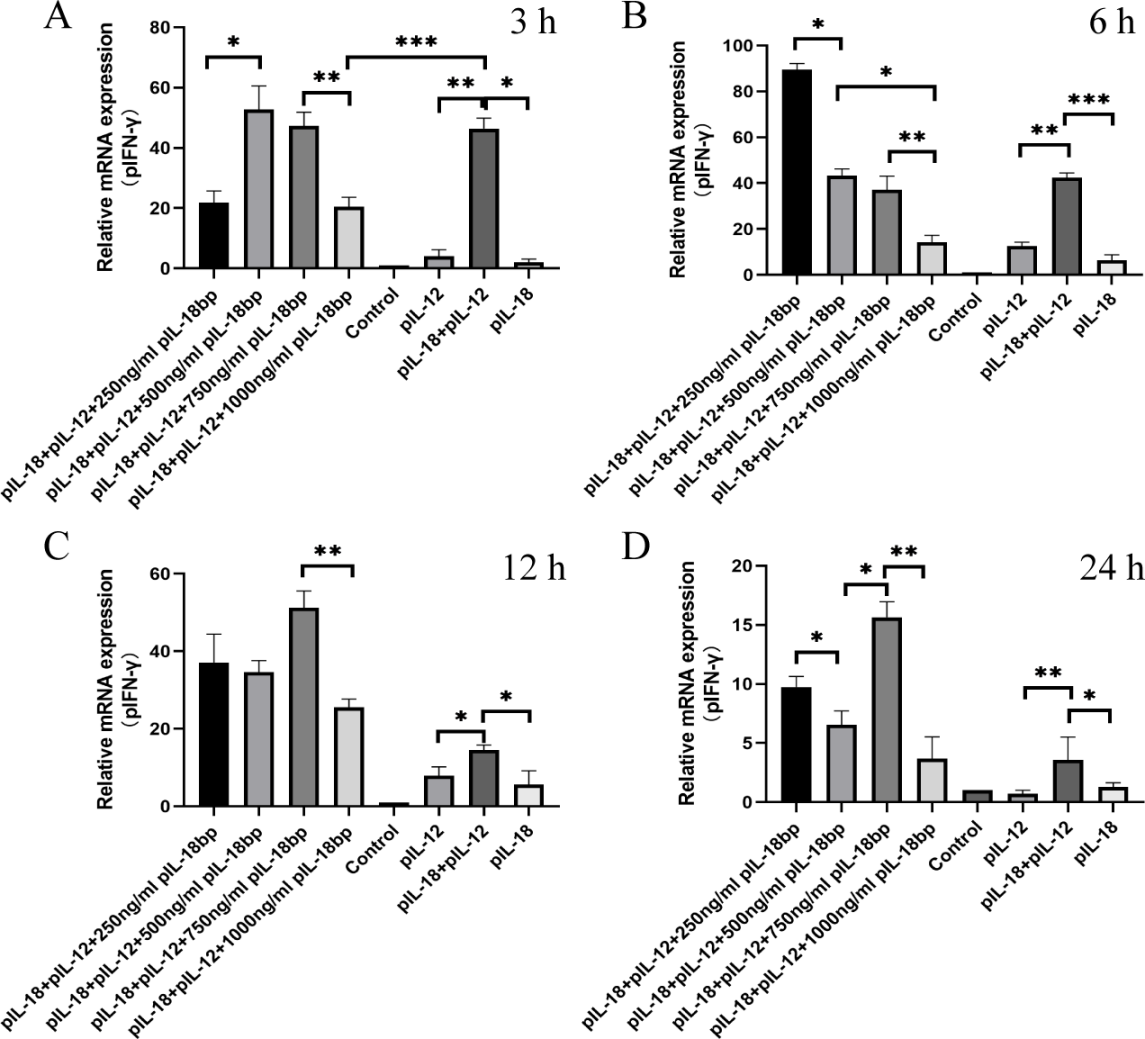
Effect of different concentrations of pIL-18BP protein on pIFN-γ mRNA expression. (**A**) Add protein for 3 h. (**B**) Add protein for 6 h. (**C**) Add protein for 12 h. (**D**) Add protein for 24 h. The data were expressed as mean ± SD. Asterisks indicate the statistical significance: *P, 0.05; **P, 0.01; ***P, 0.001.

When the concentration of pIL-18BP protein increased from 250 ng/mL to 500 ng/mL, the mRNA expression of pIFN-γ tended to increase (*P* < 0.05). However, when the concentration of pIL-18BP protein increased to 750 ng/mL and 1,000 ng/mL, the mRNA expression of pIFN-γ was significantly reduced (*P* < 0.01) (Fig. 5A). The mRNA expression of pIFN-γ was significantly lower (*P* < 0.001) at a pIL-18BP protein concentration of 1,000 ng/mL compared to the pIL-18 +pIL-12 group. This demonstrates that pIL-18BP can promote the expression of IFN-γ mRNA at low concentrations, while significantly inhibiting its expression at high concentrations (Fig. 5A). Further analysis at 6 h, 12 h, and 24 h also revealed that pIL-18BP regulated the transcript levels of pIFN-γ with a dynamic distribution (Fig. 5B, C and D). Moreover, we also found that the addition of pIL-18 or pIL-12 protein alone did not induce as high of an expression of pIFN-γ mRNA as the co-addition of pIL-18 and pIL-12 proteins (*P* < 0.05). This validates the results of a previous study which showed that pIL-12 proteins can promote pIL-18-induced expression of pIFN-γ.

### Expression of pIL-18, pIL-18BP, and pIFN-γ in peripheral blood of pigs in different PRRSV infection states

**Fig 6:**
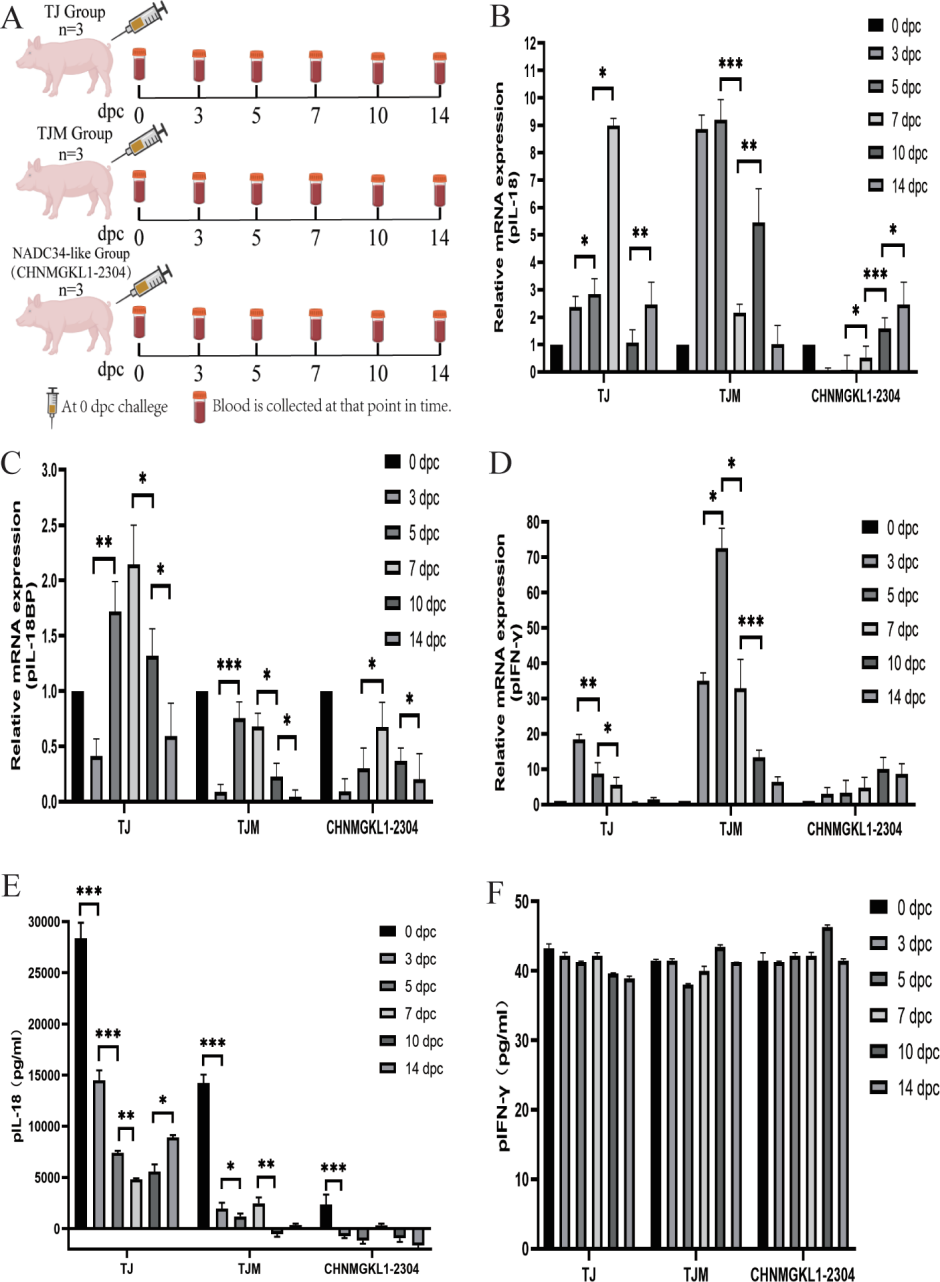
Animal experimental design and cytokine detection. (**A**) Animal testing design program. (**B**) Relative expression results of pIL-18 gene. (**C**) Relative expression results of pIL-18BP gene. (**D**) Relative expression of the IFN-γ gene. (**E**) Expression of IL-18 protein. (**F**) Expression of pIFN-γ protein. The data were expressed as mean ± SD. Asterisks indicate the statistical significance: *P, 0.05; **P, 0.01; ***P, 0.001.

The animal testing process is shown in Fig. 6A. Using 0 dpc from each group as a reference sample, we found that the mRNA expression of the pro-inflammatory factor pIL-18 gradually increased in the TJ and TJM groups after infection with the virus, and was significantly elevated in the TJ group from 3 dpc to 5 dpc to 7 dpc (*P* < 0.05). It reached a peak at 7 dpc, which was 8.9-fold higher than the expression at 0 dpc (Fig. 6B). Similarly, the TJM group had the same trend of change, with upregulated expression at 3 and 5 dpc and downregulated expression at 7 dpc (*P* < 0.001) (Fig. 6B). However, unlike the first two groups, the CHNMGKL1-2304 group showed rapid downregulation of expression after the challenge. This strain clearly suppressed the expression of pIL-18 mRNA and slowly upregulated its expression at 5, 7, 10, and 14 dpc (*P* < 0.05). Its expression at 14 dpc was 2.5-fold higher than the expression at 0 dpc (Fig. 6B). At the protein level, we were surprised to find that the TJ group had high pIL-18 protein expression at 0 dpc and showed a rapid decrease (*P* < 0.001) at 3 dpc (Fig. 6E). It reached the lowest level at 7 dpc (*P* < 0.01) and gradually increased the expression at 10 and 14 dpc (Fig. 6E). The TJM group also had the same phenomenon, with rapid downregulation of expression at 3 dpc (*P* < 0.001), reaching the lowest level at 5 dpc (*P* < 0.05), and slow upregulation of expression at 7 dpc (Fig. 6E). However, there was then a rapid downregulation of expression at 10 dpc (*P* < 0.01). Meanwhile, we were surprised to find that TJ, TJM, and NADC34-like strains infected piglets with pIL-18 were always in a state of inhibition of their protein expression compared to 0dpc (Fig. 6E). One of the NADC34-like strains resulted in protein secretion levels of pIL-18 that were consistently at the lower limit of detection of the kit (Fig. 6E).

The expression of pIL-18BP mRNA was suppressed in different groups 3 days after the challenge, and the expression gradually elevated at subsequent times (Fig. 6C). The pIL-18BP mRNA expression in the TJM and NADC34-like groups was always suppressed compared to 0dpc (Fig. 6C). The mRNA expression of pIFN-γ was upregulated in animal organisms after challenge by different PRRSV strains (Fig. 6D). Among them, the expression of pIFN-γ mRNA in the CHNMGKL1-2304 group was much less than that in the first two groups, and there was a certain promotion of mRNA expression after the challenge, but the overall effect was not obvious (Fig. 6D). The occurrence of this phenomenon may be due to NADC34-like strain specificity.

On the contrary, at the level of pIFN-γ protein, the groups had little effect on pIFN-γ protein secretion after different PRRSV challenges, and there was no significant difference between the test group and the control group (Fig. 6F). The appearance of this phenomenon is important evidence of weak and late cellular immune response after PRRSV infection in pigs.

## DISCUSSION

PRRSV is one of the most economically devastating swine pathogens, so it has attracted a great deal of attention. Currently, PRRSV elicits a weak cellular immune response and the mechanisms of its immunosuppression are not fully elucidated. IL-18 induces IFN-γ expression, but it is regulated by IL-18bp. And hIL-18BP plays a crucial role in the immune system’s response to various diseases. However, the mechanism of cellular immunomodulation induced by pIL-18BP during PRRSV infection is unclear and needs to be elucidated urgently.

In this study, we focused on the significant increase (*P* < 0.05) in pIL-18BP mRNA gene expression observed in PAM infected with the TJM strain compared to the TJ strain, as identified in the pre-RNA-seq transcriptomics analysis. To investigate further, we extracted RNA from PAM inoculated with the TJM strain and reverse-transcribed it into cDNA for use as a template. Subsequently, we designed primers based on the predicted pIL-18BP gene published by NCBI and performed PCR to amplify the pIL-18BP gene. The resulting transcripts of the five correctly sequenced pIL-18BP genes were then submitted to GenBank. Since there is no actual pIL-18BP gene in the GenBank database, we initially used primers designed for the human IL-18BP gene in this study. However, sequencing revealed that the PCR-amplified bands were actually other porcine genome sequences, not the pIL-18BP gene. We then found the predicted pIL-18BP gene in the GenBank database and used it as a template to design new primers. Using these primers, we successfully amplified bands of different sizes, which were subsequently sequenced, revealing five different transcripts.

This study marks the first time that the validated pIL-18BP gene was obtained from PAM providing a solid foundation for future research. Among the five transcripts, we selected pIL-18BP-X2, which has the longest coding region, for further investigation. We aimed to validate the pre-RNA-seq results and examine the effect of pIFN-γ on pIL-18BP. To achieve this, we identified the differences in pIL-18BP and IFN-γ mRNA expression between PAM infected with TJ and TJM strains or PBMC infected with these viruses. Interestingly, we found that the TJM group exhibited higher expression of both pIL-18BP and IFN-γ compared to the TJ group, in both PAM and PBMC. This result confirms our preliminary RNA-seq findings and demonstrates that the elevated expression of pIL-18BP may be induced by pIFN-γ.

pIL-18BP may interact with pIL-18, thereby regulating pIFN-γ mRNA expression. To validate this, the eukaryotic expression plasmids pCAGGS-pIL-18 and pcDNA3.13×flag-pIL-18BP were co-transfected into Vero E6 cells for IFA validation. It was found that both pIL-18 and pIL-18BP proteins were expressed in the cytoplasm, and their spatial encounters indicated potential interaction. In addition, a bimolecular fluorescence complementation assay was carried out, which revealed green fluorescence in the cytoplasm of the test group, confirming the interaction between pIL-18BP and pIL-18 proteins. To further observe the interaction, the CO-IP technique was employed. The results showed that the beads using the anti-flag tag successfully captured pIL-18BP and pulled down pIL-18, while the control group did not exhibit these outcomes. This confirms the direct interaction between the pIL-18BP protein and the pIL-18 protein. The presence of an important binding site between lysine (L) on pIL-18 and phenylalanine (F) on IL-18bp supports the existence of this interaction (15).

To observe the effect of the amount of pIL-18BP protein on pIFN-γ secretion, our study is based on the findings of Yan et al. regarding IL-18BP in giant pandas (25). *In vitro* experiments were conducted using the expressed protein to verify the effect of different concentrations of pIL-18BP protein on pIFN-γ mRNA expression. Our findings revealed that lower concentrations of pIL-18BP promoted IFN-γ mRNA expression, whereas higher concentrations inhibited it. In addition, we discovered that the co-addition of pIL-18 and pIL-12 proteins induced higher expression of pIFN-γ mRNA compared to the addition of either protein alone (*P* < 0.05), validating the previous study’s results that pIL-12 proteins can promote the elevated pIL-18-induced expression of pIFN-γ. To obtain pIL-18BP and pIL-18 proteins in natural conformation, we preferred the eukaryotic expression system in this study, but the expression amount and purification efficiency were low. So we replaced it with a prokaryotic expression system, which is mature, high protein expression, and easy to purify. At the same time, to ensure the natural conformation of the protein as much as possible, we optimized the method of prokaryotic expression.

To validate the expression of pIL-18, pIL-18BP, and pIFN-γ in different PRRSV-infected animals, piglets were infected with TJ, TJM, and NADC34 strains of PRRSV. The expressions of pIL-18, pIL-18BP, and pIFN-γ were assessed at various time points. The results showed that at the mRNA level, infection with the TJ and TJM strains led to a gradual increase in the expression of pIL-18 mRNA. However, the CHNMGKL1-2304 strain initially inhibited the expression of pIL-18 mRNA and then slowly increased it. Surprisingly, at the protein level, both the TJ and TJM groups initially inhibited the expression of pIL-18 protein but later showed a gradual increase. By contrast, the CHNMGKL1-2304 strain consistently inhibited pIL-18 protein expression.

IL-18 is a pro-inflammatory cytokine and an inducer of IFN-γ (26). Therefore, reducing pIL-18 protein levels may promote PRRSV reproduction. The expression of pIL-18BP mRNA detected in the *in vivo* assay differed from the results of the *in vitro* assay, which may be caused by two things (1). For the *in vitro* test, we collected samples after 3, 6, 12, and 24 h post-infection, while for the *in vivo* test we collected samples after 0, 3, 5, 7, 10, and 14 dpc (2). *In vitro* test samples are derived from a single type of cell, whereas *in vivo* test samples are derived from whole blood and have a more complex cellular composition than *in vitro* tests. Therefore, the mechanism behind the emergence of this result is the focus of our next research.

Patients with Wegener’s granulomatosis have high serum levels of both IL-18 and IL-18BP. However, the amount of IL-18BP is insufficient to neutralize IL-18, resulting in higher levels of free IL-18 compared to healthy individuals. As a result, these patients exhibit an inflammatory response (27, 28). It has also been shown that the expression of IL-18BP in the blood of asthma patients is 13 times higher than that of IL-18, suggesting excessive inhibition of IL-18 by IL-18BP in asthma (29). Therefore, it is important to establish a quantitative ELISA kit for pIL-18BP protein in future studies. This would allow simultaneous quantification of pIL-18 and pIL-18BP during PRRSV infection, enabling better assessment and treatment of the disease. Although pIFN-γ mRNA expression increased in the TJ, TJM, and CHNMGKL1-2304 infection states, there was no significant difference at the protein level. At the same time, there was a significant difference in pIL-18 protein expression, and the occurrence of this phenomenon may be related to the virulence of the strains.

In summary, this study identified five transcripts of the pIL-18BP gene for the first time. It was also demonstrated that this gene directly interacts with pIL-18 and regulates the expression of pIFN-γ mRNA. In addition, the changes in gene and protein levels of pIL-18, pIL-18BP, and pIFN-γ were investigated in piglets infected with different strains of viruses. These findings lay the foundation for a deeper exploration of cellular immune regulation factors and provide a more in-depth understanding of the role of pIL-18BP in the process of PRRSV infection.

## Data Availability

The data supporting the findings of this study are available from the corresponding author upon reasonable request. We have submitted five mutants to GenBank (GenBank numbers: OL677567–OL677571).

## References

[1] Wensvoort G, Terpstra C, Pol JM, ter Laak EA, Bloemraad M, de Kluyver EP, Kragten C, van Buiten L, den Besten A, Wagenaar F. 1991. Mystery swine disease in the Netherlands: the isolation of lelystad virus. Vet Q 13:121–130. doi:10.1080/01652176.1991.9694296 1835211

[2] Kuwahara H, Nunoya T, Tajima M, Kato A, Samejima T. 1994. An outbreak of porcine reproductive and respiratory syndrome in Japan. J Vet Med Sci 56:901–909. doi:10.1292/jvms.56.901 7865592

[3] Albina E. 1997. Epidemiology of porcine reproductive and respiratory syndrome (PRRS): an overview. Vet Microbiol 55:309–316. doi:10.1016/s0378-1135(96)01322-3 9220627

[4] Neumann EJ, Kliebenstein JB, Johnson CD, Mabry JW, Bush EJ, Seitzinger AH, Green AL, Zimmerman JJ. 2005. Assessment of the economic impact of porcine reproductive and respiratory syndrome on swine production in the United States. J Am Vet Med Assoc 227:385–392. doi:10.2460/javma.2005.227.385 16121604

[5] Nieuwenhuis N, Duinhof TF, van Nes A. 2012. Economic analysis of outbreaks of porcine reproductive and respiratory syndrome virus in nine sow herds. Vet Rec 170:225. doi:10.1136/vr.100101 22238201

[6] Ostrowski M, Galeota JA, Jar AM, Platt KB, Osorio FA, Lopez OJ. 2002. Identification of neutralizing and nonneutralizing epitopes in the porcine reproductive and respiratory syndrome virus Gp5 ectodomain. J Virol 76:4241–4250. doi:10.1128/jvi.76.9.4241-4250.2002 11932389 PMC155073

[7] Lunney JK, Fang Y, Ladinig A, Chen N, Li Y, Rowland B, Renukaradhya GJ. 2016. Porcine reproductive and respiratory syndrome virus (PRRSV): pathogenesis and interaction with the immune system. Annu Rev Anim Biosci 4:129–154. doi:10.1146/annurev-animal-022114-111025 26646630

[8] Yang L, Zhang YJ. 2017. Antagonizing cytokine-mediated JAK-STAT signaling by porcine reproductive and respiratory syndrome virus. Vet Microbiol 209:57–65. doi:10.1016/j.vetmic.2016.12.036 28069291 PMC7117332

[9] Luo X, Chen X-X, Qiao S, Li R, Xie S, Zhou X, Deng R, Zhou E-M, Zhang G. 2020. Porcine reproductive and respiratory syndrome virus enhances self-replication via AP-1-dependent induction of SOCS1. J Immunol 204:394–407. doi:10.4049/jimmunol.1900731 31826939 PMC6943376

[10] Kak G, Raza M, Tiwari BK. 2018. Interferon-gamma (IFN-gamma): exploring its implications in infectious diseases. Biomol Concepts 9:64–79. doi:10.1515/bmc-2018-0007 29856726

[11] Meier WA, Galeota J, Osorio FA, Husmann RJ, Schnitzlein WM, Zuckermann FA. 2003. Gradual development of the interferon-gamma response of swine to porcine reproductive and respiratory syndrome virus infection or vaccination. Virology 309:18–31. doi:10.1016/s0042-6822(03)00009-6 12726723

[12] Wesley RD, Lager KM, Kehrli ME. 2006. Infection with porcine reproductive and respiratory syndrome virus stimulates an early gamma interferon response in the serum of pigs. Can J Vet Res 70:176–182. 16850939 PMC1477926

[13] Cecere TE, Todd SM, Leroith T. 2012. Regulatory T cells in arterivirus and coronavirus infections: do they protect against disease or enhance it?. Viruses 4:833–846. doi:10.3390/v4050833 22754651 PMC3386620

[14] Zhang Y, Zhang Y, Gu W, Sun B. 2014. TH1/TH2 cell differentiation and molecular signals. Adv Exp Med Biol 841:15–44. doi:10.1007/978-94-017-9487-9_2 25261203

[15] Kim SH, Eisenstein M, Reznikov L, Fantuzzi G, Novick D, Rubinstein M, Dinarello CA. 2000. Structural requirements of six naturally occurring isoforms of the IL-18 binding protein to inhibit IL-18. Proc Natl Acad Sci USA 97:1190–1195. doi:10.1073/pnas.97.3.1190 10655506 PMC15564

[16] Harms RZ, Creer AJ, Lorenzo-Arteaga KM, Ostlund KR, Sarvetnick NE. 2017. Interleukin (IL)-18 binding protein deficiency disrupts natural killer cell maturation and diminishes circulating IL-18. Front Immunol 8:1020. doi:10.3389/fimmu.2017.01020 28900426 PMC5581878

[17] Murray DR, Mummidi S, Valente AJ, Yoshida T, Somanna NK, Delafontaine P, Dinarello CA, Chandrasekar B. 2012. beta2 adrenergic activation induces the expression of IL-18 binding protein, a potent inhibitor of isoproterenol induced cardiomyocyte hypertrophy in vitro and myocardial hypertrophy in vivo. J Mol Cell Cardiol 52:206–218. doi:10.1016/j.yjmcc.2011.09.022 22004899 PMC3246026

[18] Gönül Y, Genç A, Ahsen A, Bal A, Hazman Ö, Toktaş M, Ulu MS, Özdinç Ş, Songur A. 2016. The effects of IL-18BP on mRNA expression of inflammatory cytokines and apoptotic genes in renal injury induced by Infrarenal aortic occlusion. J Surg Res 202:33–42. doi:10.1016/j.jss.2015.12.026 27083945

[19] Gonul Y, Kazandı S, Kocak A, Ahsen A, Bal A, Karavelioglu A, Hazman O, Turamanlar O, Kokulu S, Yuksel S. 2016. Interleukin-18 binding protein pretreatment attenuates kidney injury induced by hepatic ischemia reperfusion. Am J Med Sci 352:200–207. doi:10.1016/j.amjms.2016.04.012 27524219

[20] Zhou T, Damsky W, Weizman OE, McGeary MK, Hartmann KP, Rosen CE, Fischer S, Jackson R, Flavell RA, Wang J, Sanmamed MF, Bosenberg MW, Ring AM. 2020. IL-18BP is a secreted immune checkpoint and barrier to IL-18 immunotherapy. Nature 583:609–614. doi:10.1038/s41586-020-2422-6 32581358 PMC7381364

[21] Iannello A, Samarani S, Allam O, Jenabian MA, Mehraj V, Amre D, Routy JP, Tremblay C, Ahmad A. 2017. A potentially protective role of IL-18 binding protein in HIV-infected long-term non-progressors. Cytokine 90:96–99. doi:10.1016/j.cyto.2016.10.018 27863336

[22] Zhang J, Sun P, Gan L, Bai W, Wang Z, Li D, Cao Y, Fu Y, Li P, Bai X, Ma X, Bao H, Chen Y, Liu Z, Lu Z. 2017. Genome-wide analysis of long noncoding RNA profiling in PRRSV-infected PAM cells by RNA sequencing. Sci Rep 7:4952. doi:10.1038/s41598-017-05279-z 28694521 PMC5504012

[23] Li Y, Mateu E, Díaz I. 2021. Impact of cryopreservation on viability, phenotype, and functionality of porcine PBMC. Front. Immunol 12:765667. doi:10.3389/fimmu.2021.765667 34912338 PMC8666977

[24] Wang FX, Liu X, Wu H, Wen YJ. 2021. Transcriptome sequencing analysis of porcine alveolar Macrophages infected with PRRSV strains to elucidate virus pathogenicity and immune evasion strategies. Virusdisease 32:559–567. doi:10.1007/s13337-021-00724-0 34631980 PMC8473512

[25] Yan Y, Deng J, Niu L, Wang Q, Yu J, Shao H, Cao Q, Zhang Y, Tan X. 2016. Cloning and characterization of giant panda (Ailuropoda melanoleuca) IL-18 binding protein. Res Vet Sci 106:170–172. doi:10.1016/j.rvsc.2016.04.004 27234556 PMC7111782

[26] Dinarello CA, Novick D, Kim S, Kaplanski G. 2013. Interleukin-18 and IL-18 binding protein. Front Immunol 4:289. doi:10.3389/fimmu.2013.00289 24115947 PMC3792554

[27] Novick D, Schwartsburd B, Pinkus R, Suissa D, Belzer I, Sthoeger Z, Keane WF, Chvatchko Y, Kim SH, Fantuzzi G, Dinarello CA, Rubinstein M. 2001. A novel IL-18BP ELISA shows elevated serum IL-18BP in sepsis and extensive decrease of free IL-18. Cytokine 14:334–342. doi:10.1006/cyto.2001.0914 11497494

[28] Mazodier K. 2005. Severe imbalance of IL-18/IL-18BP in patients with secondary hemophagocytic syndrome. Blood 106:3483–3489. doi:10.1182/blood-2005-05-1980 16020503 PMC1895045

[29] Zhang H, Wang J, Wang L, Xie H, Chen L, He S. 2018. Role of IL-18 in atopic asthma is determined by balance of IL-18/IL-18BP/IL-18R. J Cellular Molecular Medi 22:354–373. doi:10.1111/jcmm.13323 PMC5742687 28922563

